# Functional Significance of Conserved Cysteines in the Extracellular Loops of the ATP Binding Cassette Transporter Pdr11p

**DOI:** 10.3390/jof7010002

**Published:** 2020-12-22

**Authors:** Lyubomir Dimitrov Stanchev, Magdalena Marek, Feng Xian, Mara Klöhn, Daniele Silvestro, Gunnar Dittmar, Rosa Laura López-Marqués, Thomas Günther Pomorski

**Affiliations:** 1 Department of Molecular Biochemistry, Faculty of Chemistry and Biochemistry, Ruhr University Bochum, 44801 Bochum, Germany; lds@plen.ku.dk (L.D.S.); Mara.Kloehn@ruhr-uni-bochum.de (M.K.); 2 Department of Plant and Environmental Sciences, University of Copenhagen, Thorvaldsensvej 40, DK-1871 Frederiksberg, Denmark; mmarek@mpipz.mpg.de (M.M.); daniele.silvestro@hig.se (D.S.); rlo@plen.ku.dk (R.L.L.-M.); 3 Proteomics of Cellular Signaling, Luxembourg Institute of Health, Rue Thomas Edison 1A-B, L-1445 Strassen, Luxembourg; Feng.Xian@lih.lu (F.X.); Gunnar.Dittmar@lih.lu (G.D.)

**Keywords:** ABC transport proteins, sterol uptake, disulfide bonds, ATPase activity, protein trafficking

## Abstract

The pleiotropic drug resistance (PDR) transporter Pdr11p is expressed under anaerobic growth conditions at the plasma membrane of the yeast *Saccharomyces cerevisiae*, where it facilitates the uptake of exogenous sterols. Members of the fungal PDR family contain six conserved cysteines in their extracellular loops (ECL). For the functional analysis of these cysteine residues in Pdr11p, we generated a series of single cysteine-to-serine mutants. All mutant proteins expressed well and displayed robust ATPase activity upon purification. Mass-spectrometry analysis identified two cysteine residues (C582 and C603) in ECL3 forming a disulfide bond. Further characterization by cell-based assays showed that all mutants are compromised in facilitating sterol uptake, protein stability, and trafficking to the plasma membrane. Our data highlight the fundamental importance of all six extracellular cysteine residues for the functional integrity of Pdr11p and provide new structural insights into the PDR family of transporters.

## 1. Introduction

ATP binding cassette (ABC) transporters comprise a superfamily of proteins that mediate ATP-driven unidirectional transport of a variety of molecules across biological membranes. Proteins in this superfamily share a similar domain organization, with two transmembrane domains (TMD) and two cytoplasmic nucleotide-binding domains (NBDs). These four domains are often expressed as separate subunits in prokaryotic ABC proteins. In eukaryotes, ABC proteins are organized either as full transporters comprising all four domains or as “half-transporters” with a single TMD and NBD that operate as homo- or heterodimers. Among the seven eukaryotic ABC subfamilies (named A through G), ABC subfamily G (ABCG) includes both half transporters and pleiotropic drug resistance (PDR) full transporters. Within the PDR subfamily, two *Saccharomyces cerevisiae* ABC transporters, Pdr11p and Aus1p, operate as inward-directed transporters and facilitate the uptake of exogenous sterols, which is required for growth under anaerobic conditions [1,2,3,4].

**Figure 1:**
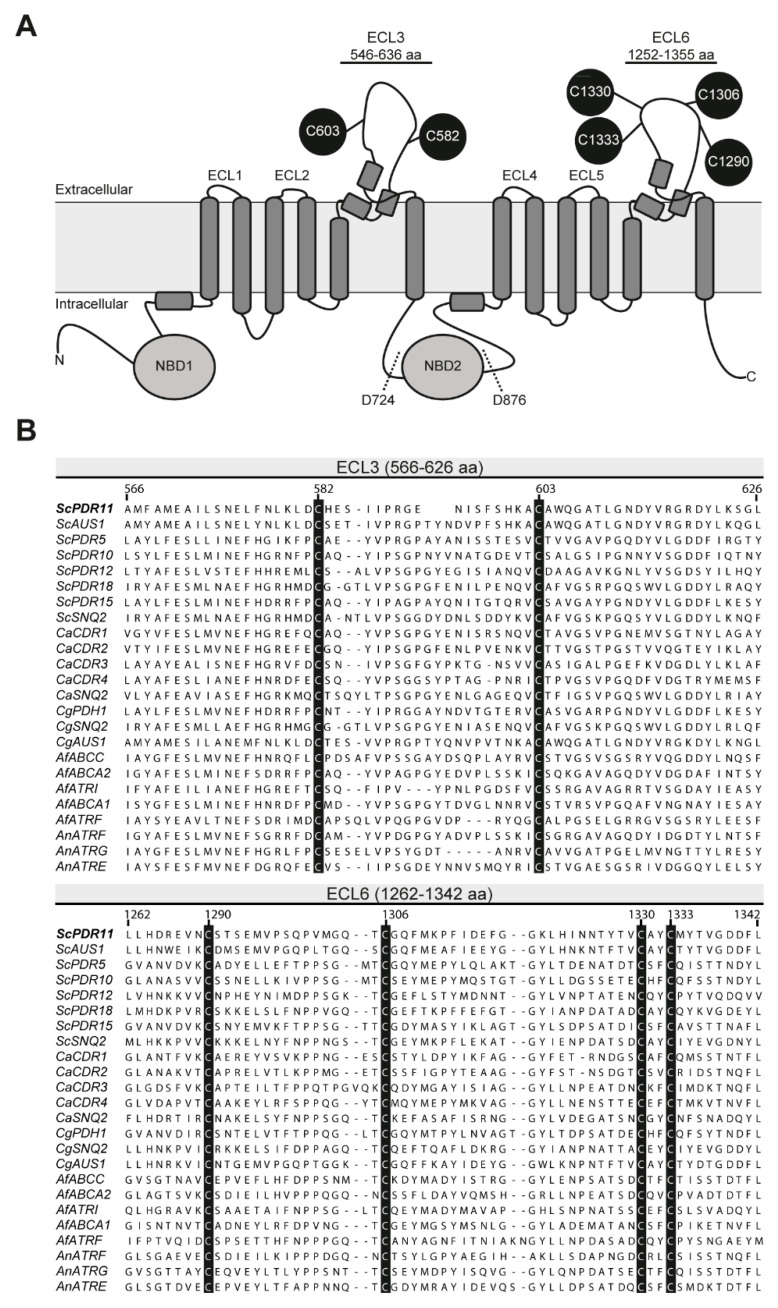
Structural features of Pdr11p. (**A**) The topological model of Pdr11p showing two transmembrane domains (TMD), each comprising six transmembrane helices, preceded by a nucleotide-binding domain (NBD). Cysteine residues in the two large extracellular loops, ECL3 and ECL6, are highlighted in black. Dashed lines indicate the recognition sites of caspase-1 at positions D724 and D876. (**B**) Partial amino acid sequence alignment of ECL3 and ECL6 from fungal ABC transporters. Conserved cysteine residues are indicated in black boxes. The transporters shown are from *S. cerevisiae* (*Sc*PDR11, *Sc*AUS1, *Sc*PDR10, *Sc*PDR15, *Sc*PDR5, *Sc*PDR12, *Sc*PDR18 and *Sc*SNQ2), *Candida albicans* (*Ca*CDR1, *Ca*CDR2, *Ca*CDR3, *Ca*CDR4, *Ca*SNQ2), *Candida glabrata* (*Cg*PDH1, *Cg*SNQ2, *Cg*AUS1), *Aspergillus fumigatus* (*Af*ABCC, *Af*ABCA2, *Af*ATRI, *Af*ABCA1, *Af*ATRF) and *Aspergillus nidulans* (*An*ATRF, *An*ATRG, *An*ATRE). Accession numbers are listed in material and methods.

Similar to other members of the PDR subfamily, Pdr11p has two major ABC transporter domains, arranged in NBD1-TMD1-NBD2-TMD2 orientation (Figure 1A). Each TMD consists of 6 TM helices. The NBDs of Pdr11p contain all characteristic sequence motifs of ABC transporters. These include Walker A and Walker B motifs (which are involved in ATP binding and hydrolysis) and the C signature sequence, a hallmark of the ABC family. The transporter has two large extracellular loops, ECL3 (91 amino acid residues) and ECL6 (104 amino acid residues), that connect the last two helices of each TMD. ECL3 and ECL6 contain two and four cysteine residues, respectively (Figure 1A). Amino acid sequence alignment highlights that these cysteine residues are highly conserved among a number of members of the fungal PDR subfamily of transporters, including Pdr11p (Figure 1B). Alanine scanning-mutagenesis of 23 cysteine residues in the multidrug ABC transporter Cdr1p from *Candida albicans* has previously shown that five cysteine residues in the intracellular loops and TMD2 of this transporter play a role in protein functionality and localization. On the other hand, the alanine-to-cysteine substitution of another six cysteine residues in the extracellular loops of Cdr1p did not affect proper trafficking and drug transport [5]. Intriguingly, in the half-size mammalian drug transporter ABCG2, a prerequisite for dimerization of a functional protein unit is the formation of an intermolecular disulfide bond between C603 in the two large extracellular loops of the homodimers. Additionally, each large extracellular loop is stabilized by the formation of an intramolecular disulfide bond between C592 and C608 [6,7].

Up to date the three-dimensional structure of Pdr11p and its substrate-binding site(s) are unknown. More structural information of Pdr11p would allow a better understanding of the molecular mechanisms of its function. In this study, we applied a combined strategy based on site-directed mutagenesis, protein purification, mass spectrometric analysis, and cell-based assays to determine the structural and functional role of conserved extracellular loop cysteines in the yeast sterol transporter Pdr11p.

## 2. Materials and Methods

*Strains, Media, and Growth Conditions.* Yeast strains and plasmids used are listed in Supplementary Tables S1 and S2. Cells were grown at 30 °C in standard synthetic dextrose (SD) or galactose (SG) medium supplemented with appropriate auxotrophic components. When necessary, the medium was supplemented with 20 μg mL^−1^ δ-aminolevulinic acid (ALA). Medium supplemented with sterols contained 0.1% (*w/v*) Tween 80 and 20 μg mL^−1^ sterols. For solid media, 2% (*w/v*) agar was added. Cells were transformed according to the lithium acetate method [8]. For growth assays, transformants were pre-cultured in SD medium supplemented with 20 μg mL^−1^ ALA, washed twice with phosphate-buffered saline (PBS; 130 mM NaCl, 2.6 mM KCl, 7 mM Na_2_HPO_4_, 1.2 mM KH_2_PO_4_, pH 7.4) or water and diluted to 0.2 optical density at 600 nm (OD_600_). Drops (3 μL) of serial 5-fold dilutions were spotted on plates and incubated at 30 °C for 5 days. To test protein stability, protein synthesis was blocked by adding the translation inhibitor cycloheximide (20 mg mL^−1^ stock in ethanol) at a final concentration of 200 μg mL^−1^ to cells grown at 30 °C to the late logarithmic growth phase and diluted in SD media to OD_600_ = 1. After cycloheximide treatment, 2 mL of cells were collected at 0, 60, 120, 180, and 240 min. Lysates from each time point were subjected to SDS-PAGE followed by fluorescence analyses.

**Figure 2:**
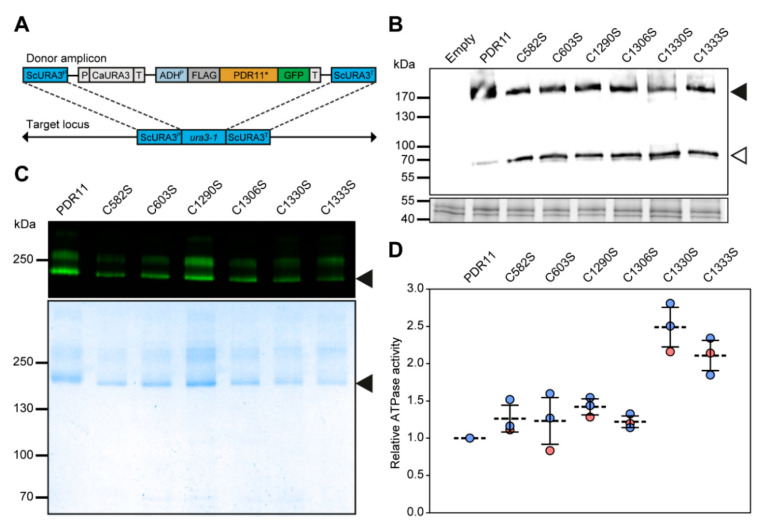
Expression and purification of Pdr11p cysteine mutants. (**A**) Genomic integration of a *FLAG-PDR11-GFP* cassette containing the *URA3* gene from *Candida albicans* (*Ca*URA3) at the deleted *URA3* locus in *hem1∆aus1∆pdr11∆* cells. Integration was achieved by homologous recombination between the *URA3* promoter (URA3P) and terminator (URA3T) regions [10]. P, promoter; T, terminator; *, *pdr11C582S*, *pdr11C603S*, *pdr11C1290S*, *pdr11C1306S*, *pdr11C1330S*, *pdr11C1333S*. (**B**) Immunoblot (upper panel) illustrating the plasmid-based expression of FLAG-tagged wild-type and mutant Pdr11p-GFP (filled arrowhead) and breakdown product (open arrowhead). Cells transformed with empty plasmids were used as a control. Blots were probed with anti-FLAG tag antibody. In-gel fluorescence detection (lower panel) using Criterion TGX stain-free gels served as the loading control. (**C**) SDS–PAGE gel representative of at least two purifications of the Pdr11p-GFP and mutant variants first analyzed by in-gel fluorescence (upper panel) and then by Coomassie blue staining (lower panel). (**D**) ATPase activity of purified Pdr11p-GFP and mutant variants was assayed as described in materials and methods using [γ-32P] ATP. Results are the means ± SD of triplicates from two independent purifications (red symbols, 1st purification; blue symbols, 2nd purification) and expressed relative to the value obtained for wild-type Pdr11p. Background signal for each experiment was subtracted from all sample measurements before calculation. A ratio of 1 corresponds to 0.25 ± 0.08 µmol/min/mg. The ATPase-inactive mutant Pdr11pK788M showed very low background ATPase activity (7 ± 2% relative to wild type, [9]).

*Plasmid Construction.* Primers used are listed in Supplementary Table S3. All polymerase chain reactions (PCRs) were carried out using Phusion^®^ High-Fidelity DNA Polymerase (New England Biolabs) following the manufacturer’s instructions. Pdr11p cysteine mutants (C582S, C603S, C1290S, C1306S, C1330S, and C1333S) were generated using the PCR-based site-directed mutagenesis kit (Stratagene, La Jolla, CA, USA) with plasmid pESC-FLAG-PDR11-GFP [9] as a template. Single integration vectors (pSIV, [10]) were generated by PCR amplification of the *FLAG-PDR11-GFP* cassette from the pESC-based template using primers containing homologous sequences to the *Bam*HI restriction site of the target pVW126 plasmid (kindly provided by Victoria Wosika, University of Lausanne, Switzerland). The *ADH1* constitutive promoter was amplified by PCR from plasmid pSIV340 using primers containing homologous regions to the *Sac*I restriction site of the target pVW126 plasmid and the FLAG-tag of the *FLAG-PDR11-GFP* cassette, respectively. Subsequently, the *FLAG-PDR11-GFP* and the *ADH1* PCR fragments were simultaneously cloned in the *Bam*HI/*Sac*I linearized single-integration vector pVW126 using the InFusion cloning kit (Takara, Mountain View, CA, USA). The resulting vectors were linearized by restriction digestion with *Pac*I and transformed into the *hem1Δaus1Δpdr11Δ* strain for integration into the *URA3* locus (Figure 2A). This strategy was also employed for the Pdr11p cysteine mutants. All integrations were verified by PCR.

*FLAG Affinity Purification and Mass Spectrometry Sample Preparation.* FLAG-affinity purification was performed essentially as described [9], with the following modifications: cells were grown in a non-selective SD medium; the lysis buffer (100 mM NaCl, 20 mM HEPES-HCl, pH 7.4) contained PhosSTOP (Roche Applied Science, Penzberg, Germany), 0.25 mM phenylmethylsulfonyl fluoride and protease inhibitor mix (1 μg/mL aprotinin, 1 μg/mL leupeptin, 1 μg/mL pepstatin, 5 μg/mL antipain and 157 μg/mL benzamidine) and solubilized membranes were incubated during affinity purification under end-over-end rotation overnight at 4 °C. The purified protein was first alkylated with iodoacetamide (20 mM final concentration) in lysis buffer (pH 7.4) supplemented with 1% *w/v n*-dodecyl β-D-maltoside (DDM) for 1 h in the dark at room temperature to block all free thiol groups on cysteine residues. Next, samples were mixed with SP3 beads (Fisher Scientific), acidified with formic acid (pH ~2), and one volume of acetonitrile was added, followed by incubation at room temperature for 10 min [11]. Upon removal of the supernatant using a magnetic rack, beads were rinsed sequentially with 70% ethanol (200 µL) and acetonitrile (180 μL) and incubated for 1 h at 37 °C in 20 μL digestion buffer (50 mM ammonium bicarbonate, 1% *w/v* DDM, pH 8) supplemented with 5 mM dithiothreitol (DTT). A second alkylation was performed with 20 mM acrylamide in the dark for 1 h at room temperature. Proteins were digested with 1 μg of trypsin (Promega, Mannheim, Germany) for 4 h at 37 °C followed by the addition of 1 μg trypsin overnight. After digestion, acetonitrile was added to a final concentration of 95%, and samples were incubated for 10 min at room temperature. Upon removal of the supernatant using a magnetic rack, beads were rinsed with acetonitrile (180 μL), resuspended in LC-MS grade water (20 μL), and incubated for 5 min at room temperature to elute digested peptides.

*Tandem Mass Spectrometry and Data Processing.* Eluting peptides were acidified with formic acid (pH < 3) and analyzed on a Q-Exactive Plus mass spectrometer (Thermo Fisher) connected to a Dionex Ultimate 3000 HPLC system (Thermo Fisher). Peptides were loaded onto a trap column (Acclaim PepMap 75 μm × 2 cm, C18, 3 μm) and separated on a 15 cm Acclaim Pepmap RSLC column (75 μm × 15 cm, C18, 2 μm) using an 89 min gradient (2% to 90% acetonitrile) with a flow rate of 0.3 μL/min. MS data were acquired in either a data-dependent mode (DDA) or a targeted mode (PRM). In DDA acquisition, MS1 resolution of 70,000 and a scan range from 375 to 1500 *m*/*z* with AGC target of 3 × 10^6^ were used. The top 12 abundant peaks from the MS1 scan were selected for fragmentation with MS2 resolution of 17,500 and maximum injection time of 45 ms, and an isolation window of 1.4 *m*/*z* and AGC target of 10^5^ for fragments were set. Dynamic exclusion of 20 s and normalized collision energy of 28 were specified. In PRM experiments, the masses of targeted peptides are shown in Supplementary Table S4. The key parameters in PRM acquisition were set as 35,000 resolution for MS2 scan, 3 × 10^6^ for AGC target, and 120 ms for maximum injection time. Each sample was analyzed in triplicates. The MS raw data (DDA) were searched against a customized proteome database (Pdr11p sequence with 1000 human proteins) in PEAKS Studio software from Bioinformatics Solutions, Inc. (Waterloo, ON, Canada) with carbamidomethylation and propionamide as variable modifications on cysteine. The false discovery rate of matched peptides was set to 5%. Data from PRM experiments were loaded to Skyline software [12] for quantitative analysis. The sum area of selected product ions was used for quantitative comparison.

*ATPase Assay.* ATPase activity was measured at 28 °C for 60 min as described previously using 1 mM ATP, 5 mM MgCl_2_, 5% *w/v* DDM, and 2 μCi of [γ-^32^P] ATP [9,13]. The release of inorganic phosphate was determined by indirect β-counting (Perkin Elmer 1450 MicroBeta Trilux, Wallac).

*Membrane Fractionation.* Cells expressing genomically integrated wild-type and mutant variants of Pdr11p-GFP were harvested (850× *g*, 5 min, 4 °C), washed twice in ice-cold PBS, and lysed by vortexing with glass beads at 50 OD_600_ mL^−1^ in gradient buffer (50 mM HEPES-KOH, pH 7.2, 10 mM EDTA, 0.8 M sorbitol) supplemented with 1 mM phenylmethylsulfonyl fluoride, 2 mM N-ethylmaleimide and protease inhibitor mix. Subcellular membranes were collected on a sucrose cushion (60% *w/w*; 200 µL) at 100,000× *g* (60 min, 4 °C) and loaded on top of a sucrose gradient prepared in gradient buffer using the following steps: 0.5 mL 60%, 1 mL 40%, 1 mL 37%, 1 mL 35%, 1 mL 30% (*w/w*) sucrose. After centrifugation (130,000× *g*, 16 h, 4 °C, SW55Ti), 10 × 0.5 mL fractions were collected from the top. Equal volumes per fraction were used for western blot analysis.

*SDS PAGE and Western Blot Analysis.* Protein samples were separated on 10% SDS-PAGE gels or Criterion TGX Stain-free 4–15% Tris-Glycine protein gels (Bio-Rad Laboratories, Hercules, CA, USA), transferred to nitrocellulose membranes (Merck Millipore, Milford, MA, USA), and immunodetected with mouse monoclonal antibodies against FLAG (1:2000; Sigma-Aldrich, St. Louis, MO, USA), Dpm1p (1:250; Invitrogen Molecular Probes, Carlsbad, CA, USA), and Pma1p (1:3000; Thermo Fisher Scientific, Rockford, IL, USA). Protein blots were probed with horseradish peroxidase (HRP)-conjugated secondary antibodies (Dako A/S, Glostrup, Denmark), which were detected using enhanced chemiluminescence (Thermo Fisher Scientific, Rockford, IL, USA). In-gel GFP fluorescence was detected with a ChemiDoc™ MP device (Bio-Rad Laboratories GmbH, München, Germany) using the Image Lab™ software and DyLight 488 channel filter for Blue Epi illumination. Purity and concentration of the purified protein were determined via densitometry analysis by Coomassie Blue staining using bovine serum albumin as a standard (ChemiDoc™ MP device). Digestion with caspase-1 (25 units, C5482; Sigma-Aldrich) of purified protein (200 ng) was performed in caspase buffer (50 mM HEPES, pH 7.2, 50 mM NaCl, 0.1% *w/v* DDM, 10 mM EDTA, 5% *v/v* glycerol and 10 mM DTT) for 4 h at 37 °C. Before analysis by SDS PAGE, samples were incubated with gentle shaking in SDS loading buffer (±50 mM DTT) for 30 min at 37 °C.

*Fluorescence Microscopy.* Fluorescence microscopy and image acquisition were carried out on living cells immobilized on Concanavalin A-coated glass slides using inverted confocal laser scanning microscopes (Leica TCS SP5 and SP8, Heidelberg, Germany). All images were acquired using a 63×/1.2 numerical aperture (NA) water objective. GFP was excited at 488 nm and emission was recorded from 500 nm to 560 nm. Acquired images were analyzed with the open-source ImageJ software (National Institutes of Health, Madison, WI, USA).

*Sterol Uptake Assays.* For uptake of NBD-cholesterol (25-[N-[(7-nitro-2-1,3-benzoxadiazol-4-yl)methyl]amino]-27-norcholesterol; Avanti Polar Lipids, Alabaster, AL, USA), cells expressing plasmid-borne Pdr11p variants were cultured for 16 h in SG medium containing 0.1% Tween 80, 5 µg mL^−1^ NBD-cholesterol and 2.5% (*w/v*) bovine serum albumin. Cells were washed twice with PBS followed by one wash in PBS supplemented with 0.05% (*v/v*) NP-40 and analyzed by flow cytometry using a Becton Dickinson FACS Calibur^TM^ system (San Jose, CA, USA). One microliter of 1 mg mL^−1^ propidium iodide in water was added to 10^7^ cells in 1 mL water just before analysis. Twenty thousand cells were analyzed without gating during acquisition using the following fluorescence channels (in log scale): FL1 (530/30 nm, NBD-cholesterol) and FL3 (670 nm long-pass filter, propidium iodide). Data were processed with either Cyflogic (BD Biosciences, San Jose, CA, USA) or Flowing Software (Turku Centre for Biotechnology, Turku, Finland). Main cell populations were gated based on forward and side scattering values. Live cells were selected based on propidium iodide exclusion. Green fluorescence (NBD-cholesterol) of living cells was plotted on a histogram and the geometric mean of the fluorescence intensity was calculated and normalized to empty vector control. For [^14^C]cholesterol uptake, cells were cultured for 20 h in SG medium containing 0.1% *v/v* Tween 80 and 0.1 µCi mL^−1^ [^14^C]cholesterol (Hartmann Analytic GmbH, Braunschweig, Germany) and analyzed as described previously [14]. Radioactivity was measured by scintillation counting (Perkin Elmer 1450 MicroBeta Trilux, Wallac Oy, Turku, Findland). To verify equal lipid recovery, phosphatidylcholine content was measured by a colorimetric enzymatic assay (Kit MAK049; Sigma-Aldrich, St. Louis, MO, USA). Analysis of non-labeled cholesterol uptake by mass spectrometry was performed as described previously [15]. β-Sitosterol was used both as internal and as external standard in individual experiments, and sample peaks were obtained from cholesterol.

*Sequence Alignment of ABC Transporters.* Alignment of ABC transporter sequences was completed with the Clustal Omega algorithm [16] using default settings and visualized with the Jalview software [17]. Accession numbers in the UniProt database were: for *S. cerevisiae*, *Sc*Pdr11p (P40550), *Sc*Aus1p (Q08409), *Sc*Pdr10p (P51533), *Sc*Pdr5p (P33302), *Sc*Pdr12p (Q02785), *Sc*Pdr18 (P53756) and *Sc*Snq2p (P32568); for *Candida albicans, Ca*Cdr1p (P43071), *Ca*Cdr2p (P78595), *Ca*Cdr3p (O42690), *Ca*Cdr4p (O74676) and *Ca*Snq2p (A0A1D8PQ95); for *Candida glabrata Cg*Aus1p (A7VL21), *Cg*Pdh1p (O74208) and *Cg*Snq2p (Q6FQN3); for *Aspergillus fumigatus Af*ABCC (E9RBG1), *Af*ABCA2 (Q4X006), *Af*ATRI (Q4WWW3), *Af*ABCA1 (Q4WR59) and *Af*ATRF (Q4WDD4); for *Aspergillus nidulans An*ATRF (Q96VK5), *An*ATRG (Q96VK4) and *An*ATRE (Q96VK6). The sequence of Pdr11p was used as reference sequence for the alignment.

*Data Availability.* All source data for figures and tables in the presented article are available from the corresponding authors.

## 3. Results

### 3.1. Expression and Purification of Pdr11p Single Cysteine Mutants

To examine the role of ECL cysteine residues in the functioning of Pdr11p, each of the six conserved extracellular cysteine residues present in ECL3 and ECL6 (Figure 1) was replaced with serine, an amino acid of similar size and charge, using site-directed mutagenesis. The resulting mutants (C582S, C603S, C1290S, C1306S, C1330S, and C1333S) and wild-type constructs, additionally engineered with an N-terminal FLAG-tag and a C-terminal GFP, were integrated into the genome of the *hem1Δpdr11Δaus1Δ* strain under the control of the *ADH1* constitutive promoter (Figure 2A). Alternatively, all mutants and wild-type constructs were expressed from a multi-copy plasmid under the control of a galactose-inducible promoter in the *hem1Δpdr11Δaus1Δ* strain. The GFP tag has been shown not to alter the functional properties of Pdr11p and facilitated analysis of protein expression, purification, and stability using GFP fluorescence [9].

All variants were expressed as full-length proteins (FLAG-Pdr11p-GFP 189 kDa) as validated by immunoblotting of total membrane preparations (Figure 2B). An additional band of about 70 kDa was detected by the anti-FLAG antibody, indicating some proteolytic degradation, in particular for the mutant variants. Anti-FLAG affinity purification resulted in the isolation of each GFP-fused variant to apparent homogeneity (98% pure based on densitometric analysis), although not homodispersed, as judged by Coomassie staining and in-gel fluorescence (Figure 2C). The yield of protein per liter of culture varied between 40 and 100 mg, with typically lower yields for the cysteine mutants. Wild-type and cysteine mutants were functionally assayed by determining their basal ATPase activity. All purified proteins displayed a robust ATPase activity (Figure 2D).

### 3.2. Pdr11p Cysteine Mutants Show Impaired Ability to Support Cellular Sterol Uptake

**Figure 3:**
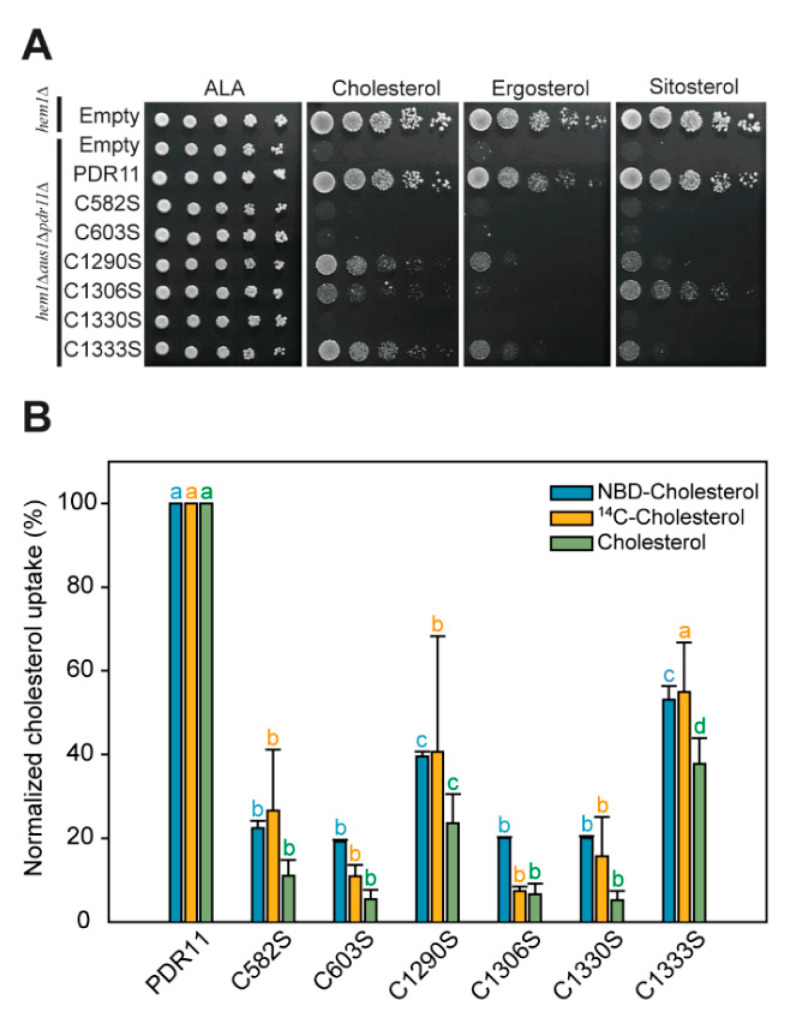
Pdr11p cysteine mutants show impaired ability to support sterol uptake. (**A**) Functional complementation of the *hem1Δaus1Δpdr11Δ* strain expressing genomically integrated wild-type and mutant variants of Pdr11p-GFP. Yeast strains *hem1Δ* and *hem1Δaus1Δpdr11Δ* transformed with empty plasmids were used as positive and negative controls, respectively. Yeast strains were serially diluted (five-fold) and grown on plates containing δ-aminolevulinic acid (ALA), cholesterol, ergosterol, or sitosterol, as indicated, for 5 days at 30 °C. The experiment was repeated twice with identical results. (**B**) Sterol transport activity of plasmid-borne Pdr11p cysteine mutants in *hem1Δaus1Δpdr11Δ* cells. Uptakes of NBD-cholesterol, [^14^C]-cholesterol, and cholesterol were measured as described in materials and methods. Results represent the means ± SD from two (unlabeled cholesterol) or three (NBD- and [^14^C]-cholesterol) independent experiments and are expressed as percentage signal intensity relative to cells expressing wild-type Pdr11p; 100% corresponds to 589.8 ± 16.5 a.u. (NBD-cholesterol), 462.1 ± 74.9 a.u. ([^14^C]-cholesterol), and 168.7 ± 54.0 µg (cholesterol). For each individual cholesterol form, statistical significance was calculated using a two-way ANOVA analysis followed by Tukey HSD. Identical letters (a, b, c, d) above bars for a specific cholesterol form indicate values that do not show any statistically significant difference (*p* < 0.01).

To evaluate the influence of the individual cysteine mutations on sterol uptake, the wild type and cysteine mutants of Pdr11p-GFP were tested for their ability to complement the *hem1Δaus1Δpdr11Δ* strain on medium containing sterols (cholesterol, ergosterol, sitosterol). Under these conditions, *hem1Δ* cells lacking the *PDR11* and *AUS1* genes display a growth defect but grow normally on a medium containing δ-aminolevulinic acid, the enzymatic product of the first step in heme biosynthesis catalyzed by aminolevulinic synthase [18]. The growth defect of the *hem1Δaus1Δpdr11Δ* strain on sterol-containing media was complemented by wild-type Pdr11p-GFP (Figure 3A), confirming the functionality of the tagged protein. Out of the six cysteine mutants, C1290S and C1333S partially rescued growth of the *hem1Δaus1Δpdr11Δ* strain on medium supplemented with cholesterol and to a lower degree on ergosterol- and sitosterol-containing media, while the C1306S mutant partially rescued growth on sitosterol- and cholesterol-supplemented media but not on ergosterol.

To further test the role of the six cysteine residues in the function of Pdr11p, uptake assays with the fluorescent sterol analog NBD-cholesterol were carried out (Figure 3B). Uptake studies with [^14^C]-cholesterol and unlabeled cholesterol served to validate the use of the fluorescent cholesterol analog. For these experiments, wild-type Pdr11p and cysteine mutants were expressed without GFP tag from a multi-copy plasmid under the control of the galactose-inducible GAL10 promoter. Expression of wild-type Pdr11p in the *hem1Δaus1Δpdr11Δ* strain resulted in significant accumulation of NBD-cholesterol, as revealed by flow cytometry analysis (Figure 3B). Mutants C582S, C603S, C1306S, and C1330S had no detectable NBD-cholesterol uptake activity, consistent with their growth phenotype. The C1290S and C1333S mutants showed around 39 ± 1 and 53 ± 3% (*n* = 3), respectively, of the wild-type activity. Similar differences for cells expressing the six cysteine mutant proteins were observed for the uptake of [^14^C]-cholesterol and unlabeled cholesterol, ruling out that the presence of the fluorescent NBD-tag has an impact on the outcome of the uptake assays.

### 3.3. Single Cysteine Mutations Affect Trafficking of Pdr11p to the Plasma Membrane and Protein Stability

**Figure 4:**
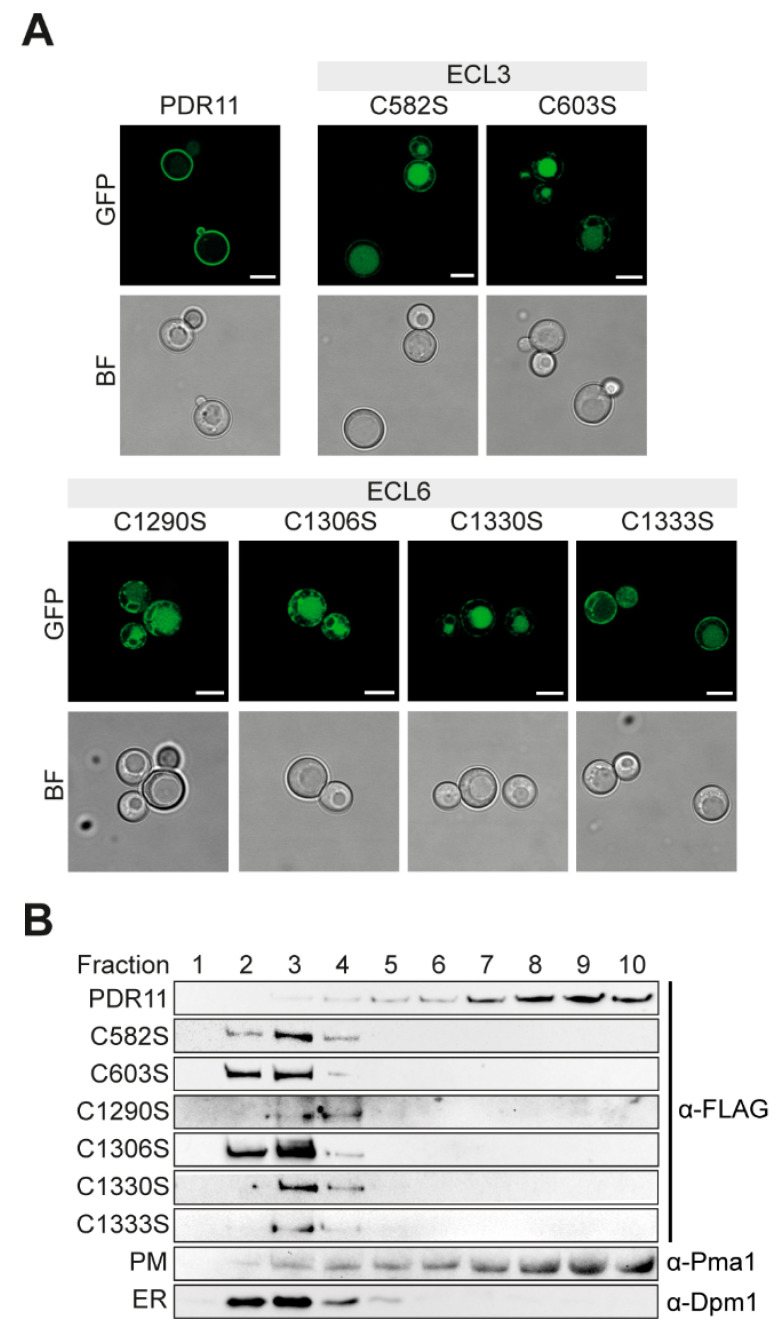
Pdr11p cysteine mutants display impaired trafficking to the plasma membrane. (**A**) *hem1Δaus1Δpdr11Δ* cells expressing genomically integrated wild-type and mutant Pdr11p-GFP were grown to the mid-logarithmic phase before imaging by fluorescence (GFP) or bright field (BF) microscopy. Scale bars, 5 µm. (**B**) Sucrose gradient fractionation of membranes prepared from *hem1Δaus1Δpdr11Δ* cells expressing genomically integrated wild type and mutant Pdr11p-GFP. Gradient fractions were assayed by immunoblotting using antibodies against the FLAG epitope engineered into Pdr11p or two organellar markers (Pma1p and Dpm1p). Shown are fractionation profiles for marker proteins corresponding to membranes of cells expressing wild-type Pdr11p-GFP, but profiles were determined individually for each membrane preparation, with similar results. PM, plasma membrane; ER, endoplasmic reticulum. Results are representative of at least two independent experiments.

The substantial reduction in sterol uptake for the six Pdr11p cysteine mutants could result from a decrease in cell surface expression. Therefore, we next studied the effect of the mutated cysteine residues on the correct targeting of the transporter to the cell surface. For this, *hem1Δaus1Δpdr11Δ* cells expressing wild-type Pdr11p-GFP or each of the six cysteine mutants were grown in a cholesterol-supplemented medium and subsequently visualized by fluorescence confocal microscopy. For wild-type Pdr11p-GFP, robust surface expression was detected (Figure 4A). In contrast, the GFP signals of the mutant proteins carrying serine substitutions of the conserved extracellular cysteine residues were barely detectable at the membrane surface but were visible in the ER and vacuole. In yeast, the ER can be recognized as a ring surrounding the nucleus connected by thin strands to cortical ER underlying the plasma membrane, and vacuoles are large round structures that appear as indentations in bright field images. To corroborate the microscopic data, all strains were subjected to subcellular fractionation to separate the plasma membrane from intracellular organelles (Figure 4B). The correct separation of cellular membranes was verified using two marker proteins, ER-resident dolichol-1-phosphate-mannose synthase 1 (Dpm1p) and the plasma membrane ATPase 1 (Pma1p). Wild-type Pdr11p-GFP displayed the most prominent signal in the fractions corresponding to the plasma membrane. By contrast, and in line with the microscopy results, all six cysteine mutants were detected exclusively in the light membrane fractions of the ER.

**Figure 5:**
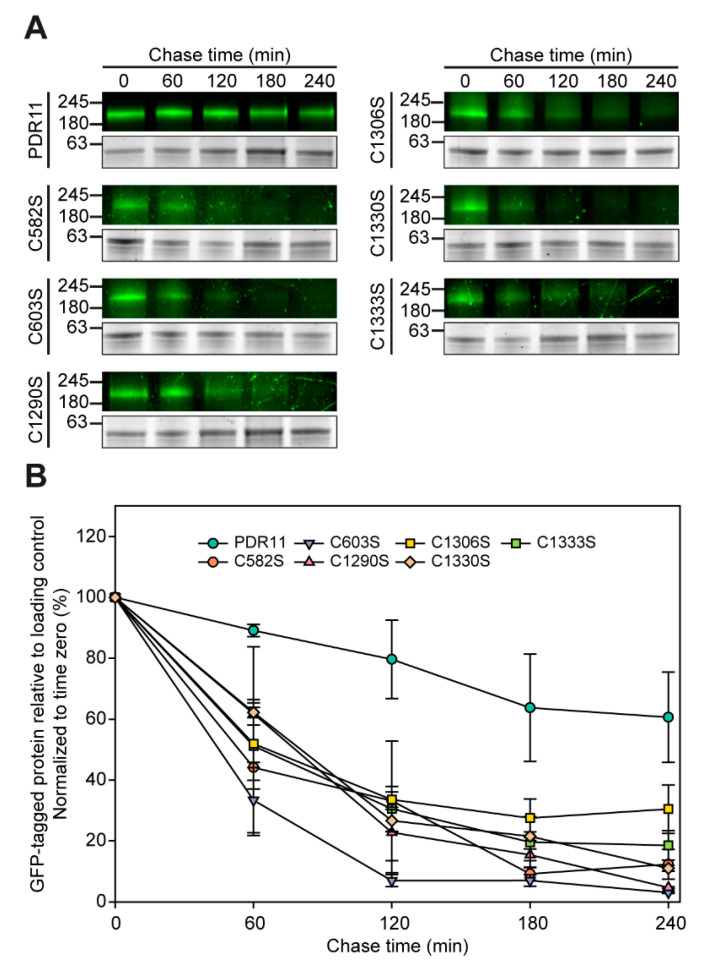
GFP-tagged Pdr11p cysteine mutants are less stable than the wild type. *hem1Δaus1Δpdr11Δ* cells expressing genomically integrated wild-type and mutant Pdr11p-GFP were treated with cycloheximide and processed at the indicated time points to monitor the stability of the ABC transporter. (**A**) Upper panels display in-gel GFP fluorescence and lower panels in-gel fluorescence detection at ~63 kDa for loading control. (**B**) The in-gel fluorescence intensities of GFP-tagged Pdr11p or cysteine mutant variants were quantified and normalized to the intensities of the loading control at ~63 kDa for each time point. Results are expressed as the percent change from time zero, which was set at 100%. Data are shown as the mean ± SD from two biological replicates.

We next investigated the stability of the six Pdr11p cysteine mutants and wild-type Pdr11p-GFP by blocking new protein synthesis with cycloheximide and analyzing the amounts of the ABC transporter variants present in the cell at different time points (Figure 5). All mutants were less stable than wild-type Pdr11p. Within 4 h, the six Pdr11p cysteine mutants showed 60 to 100% degradation, whereas wild-type Pdr11p levels decreased by approximately 40% only (Figure 5). However, protein stability did not correlate with the ability of the mutant transporters to functionally complement yeast sterol uptake mutants (Figure 3). We conclude that cysteine residues C582, C603, C1306, C1290, C1330, and C1333 in the ECLs of Pdr11p are important for proper trafficking of the protein to the plasma membrane and for stabilizing the protein structure.

### 3.4. Mapping Disulfide Bonds in the ECLs of Pdr11p

**Figure 6:**
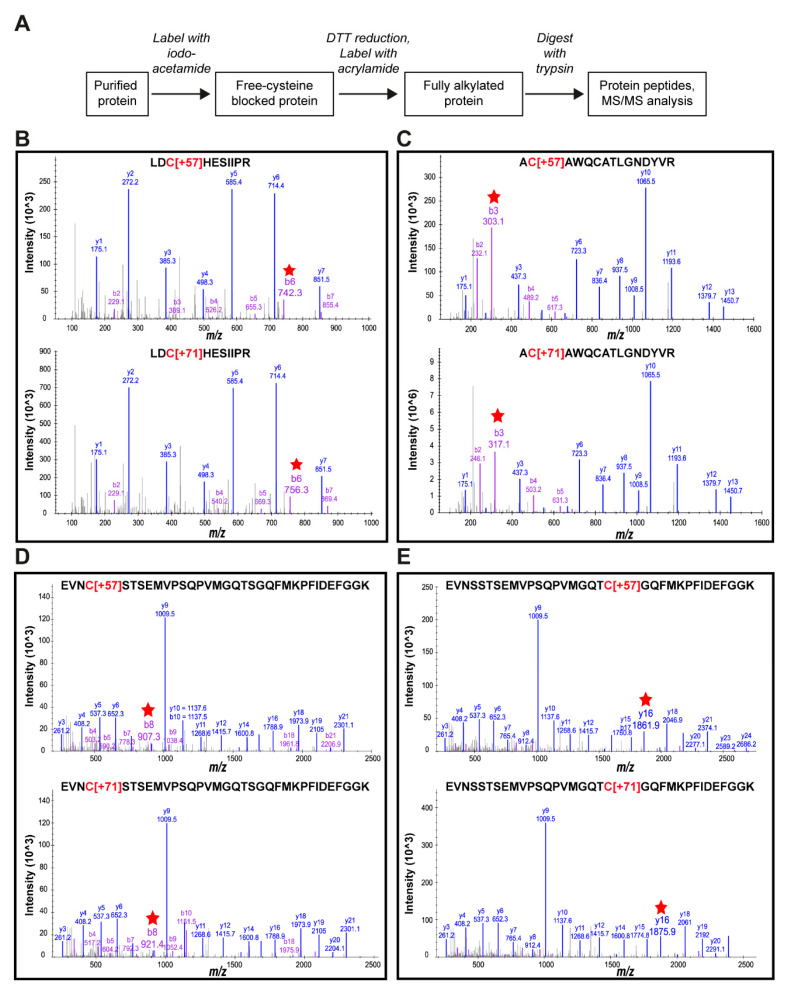
MS/MS spectra of peptide adducts obtained by a tryptic digest of Pdr11p. (**A**) Experimental flow chart for the chemical modification strategy and subsequent mass spectrometry-based identification of alkylated protein adducts. (**B**–**E**) Four cysteines (**B**, C582; **C**, C603; **D**, C1290; **E**, C1306) were identified with two kinds of adducts, indicating their potential involvement in disulfide bonds. Red arrows indicate the representative product ions with 14-Da mass differences by comparing the upper and lower spectra of each peptide.

To identify putative disulfide bonds between the cysteine residues in ECL3 and ECL6, a two-step chemical modification was performed utilizing purified wild-type and single-cysteine mutants of Pdr11p-GFP (Figure 6A). Free thiol groups in the protein were first alkylated with iodoacetamide, resulting in the coupling of a carbamidomethyl group to the free cysteine residue and a mass increase of +57-Da/thiol. After removal of the first reagent, the protein was treated with DTT to reduce any disulfide bonds present, which were subsequently blocked with acrylamide, resulting in a mass increase of +71-Da/thiol. Following tryptic digestion, peptides were identified from tandem mass spectra obtained by LC-MS/MS. For four out of six loop cysteines (C582, C603, C1290, and C1306), we were able to observe peptide adducts from both alkylation steps (Figure 6B–E). Modification by the second agent was not observed when the protein was already treated with DTT before the first alkylation step, indicating that acrylamide labeling reports on disulfide-bonded cysteines. After two-step alkylation of purified wild-type Pdr11p, about 76% of C582, 60% of C603, 68% of C1290, and 64% of C1306 were found modified with acrylamide (Table 1), suggesting their involvement in disulfide bonds. The percentage of the acrylamide-modified fraction for C582 dropped to 32% in the Pdr11p mutant C603S but not in the other mutants, and the same trend was found for C603 in the Pdr11p mutant C582S. These results are highly indicative of the presence of a disulfide bond between C582 and C603 in ECL3. Interestingly, we also observed a 20% decrease of acrylamide modification of C582 and C1290 in Pdr11p mutant C1306S, implying a potential additional disulfide bond in ECL6. To test the presence of potential disulfide bonds between the cysteine residues in ECL3 and ECL6, purified wild-type Pdr11p-GFP was subjected to limited proteolysis using caspase-1. This enzyme cleaves Pdr11p at two sites, i.e., D724 and D876 of the NBD2, resulting in a fragmentation of the full-length protein (Supplementary Figure S1). Comparisons of the migration behavior of the digested wild-type protein in SDS-PAGE under disulfide reducing and non-reducing conditions revealed identical fragments of 88, 65, and ~60 kDa MW, indicative of the absence of disulfide bonds between both ECLs. We conclude that cysteines C582 and C603 form a disulfide bond in native Pdr11p.

## 4. Discussions

In this study, the mutational analysis was used to identify functionally important extracellular cysteine residues and their involvement in disulfide bond formation in Pdr11p. The strategy included the expression of wild-type Pdr11p and its single cysteine mutants as GFP fusion proteins from genomically integrated genes, avoiding heterogeneous expression levels within the cell population typically observed for plasmid-based systems [9,14]. Each cysteine residue was individually mutated to serine and the modified proteins were expressed, purified, and characterized.

The mass spectrometric analysis was used to probe for the existence of disulfide bonding in the extracellular loops via a two-step alkylation strategy combined with dithiothreitol reduction. This approach provided clear evidence for the formation of an essential intramolecular disulfide bond between C582 and C603 in ECL3. Our mass spectrometry data further suggest the presence of additional intramolecular disulfide bond(s) within ECL6. Precise assignment of these disulfide bond(s), however, was hampered by the lack of peptide detection for two of the four cysteines and by inconclusive results for the single mutant analysis, potentially caused by the formation of non-native disulfide bonds in the mutants during protein folding and maturation. The extracellular cysteines might also exist in an equilibrium between labile disulfide bonds and free thiols, which would have been trapped during prolonged incubation with the first alkylated agent. For the human ABC transporters ABCA1 and ABCA4 interloop disulfide bonds have been identified [19,20,21]. In contrast, limited proteolysis and analysis of digestion patterns by SDS-PAGE demonstrated the absence of interloop disulfide bonds between ECL3 and ECL6 of wild-type Pdr11p.

Expression of the single cysteine mutants resulted in intracellular retention and increased degradation. This result implies that correct disulfide bond formation between the extracellular loop cysteines is important for stabilizing the protein structure. Native disulfide bonds were also found to be important for the structural integrity and cell surface trafficking in other ABC transporters. For the human transporters, ABCB6, and sulfonylurea receptor 1 (SUR1)/ABCC8, the disruption of disulfide bonds results in protein degeneration [21]. A similar stabilizing effect of an intramolecular disulfide bond was reported for the human ABC transporter ABCG2 [6,7,22,23]. It is noteworthy that the two disulfide-bond-forming cysteine residues of ABCG2, C592, and C608, are located in the ECL of the transporter at close-by positions to the cysteine residues C582 and C603 of Pdr11p. Similar to the Pdr11p residues, mutation of C592 and C608 in ABCG2 results in trafficking defects and altered functionality [6]. Moreover, removal of cysteine residues not involved in disulfide bond formation can also lead to impaired trafficking and mislocalization of the ABC transporter protein, as shown for several membrane transporters [6,24,25].

Our functional cell-based assays revealed that cells expressing any of the cysteine mutants of Pdr11p displayed impaired uptake of various sterols and sterol analogs in respect to cells expressing wild-type Pdr11p. The dramatic reduction in transport activity following mutation of individual extracellular loop cysteines in Pdr11p was most likely caused by a defect in targeting of the ABC transporter to the plasma membrane, as cell surface expression was not apparent for the single cysteine mutants. In line with this notion, all purified cysteine mutants exhibited a stable basal ATPase activity, ruling out a direct effect on the functionality of the transporter. This basal activity is within the range of values reported for a number of other purified eukaryotic ABC transporters [26,27,28,29,30]. Two purified detergent-solubilized cysteine mutants (C1330S and C1333S) exhibited ~2-fold increased basal ATP activity. The observed increase in basal activity might be caused by structural changes in the cysteine mutants resulting in reduced stripping of bound lipids during purification. It is well established that the ATPase activity of ABC transporters is affected by the lipid environment [31]. Structural changes might also affect directly the intrinsic (uncoupled) ATPase activity.

Notably, three cysteine mutants (C1290S, C1306S, and C1333S) still retained a reduced ability to transport certain sterols. Yeast strains impaired in sterol uptake but expressing Pdr11p mutants C1290S and C1333S were able to grow on media supplemented with cholesterol and to accumulate fluorescent and native cholesterol, although to a much lesser extent than cells expressing wild-type Pdr11p. Surprisingly, cells expressing Pdr11p mutant C1306 grew on plates supplemented with the plant sterol sitosterol but not on other sterols. It would be therefore interesting to test further its ability to accumulate this sterol variant. Thus, mutations within the large extracellular loop may change the substrate affinity of Pdr11p. Similarly, mutations in the disulfide-bond-forming cysteine residues of ABCG2, C592, and C608, altered the substrate specificity of the transporter [6].

A number of cell wall proteins have been proposed to serve as sterol-binding proteins that may facilitate the uptake of sterols by Pdr11p [32,33]. Conceivably, single cysteines and/or correct disulfide bond formation in the extracellular loops of Pdr11p might be important for the interaction of the transporter with cell wall proteins. Such a mechanism has been described for the human ABC transporter ABCA1 for the interaction with apolipoprotein A-I [19]. Alternatively, the disulfide bonds within the two long extracellular loops might stabilize the binding and/or translocation states of the transporter.

In conclusion, we have provided evidence that the cysteines within the two large extracellular loops of Pdr11p are critical for plasma membrane targeting and might influence the substrate affinity of the transporter. Further studies will be needed to determine the mechanism by which these cysteines affect the transport properties of Pdr11p. Given the highly conserved nature of the six cysteine residues within the fungal PDR transporter subfamily, elucidating their precise molecular function will provide an invaluable understanding for the development of new antifungal drugs.

## Figures and Tables

**Table 1:** Percentage of acrylamide-reacted fraction for individual cysteines in ECL3 and ECL6 of Pdr11p. Purified wild-type and single cysteine mutants of Pdr11p-GFP were subjected to two-step chemical modification and LC-MS/MS analysis. Dark and light blue shadowing highlights cysteines with a strong (>35%) and slight drop (>15%), respectively, in the percentage of acrylamide modification as compared to the wild-type protein. Each sample was analyzed in technical triplicates on LC-MS/MS, and the average total peak area was calculated.

T	C582 (%)	C603 (%)	C1290 (%)	C1306 (%)
Wild type	75.7	60.1	67.8	64.3
Mutant C582S	----	23.6	68.2	65.7
Mutant C603S	32.4	----	56.7	54.8
Mutant C1290S	70.4	64.1	----	62.8
Mutant C1306S	56.9	48.9	48.8	----
Mutant C1330S	65.1	62.7	64.2	69.4
Mutant C1333S	71.6	71.1	76.5	81.8

## Supplementary Materials

The following are available online at https://www.mdpi.com/2309-608X/7/1/2/s1, Table S1: Yeast strains used in this study, Table S2: Plasmids used in this study; Table S3: Oligonucleotides used in this study, Table S4: List of targeted peptides for LC-MS/MS analysis, Figure S1: Analysis of Pdr11p-GFP after limited proteolysis under reducing or non-reducing conditions.
