# Supplementary material for: Functional Significance of Conserved Cysteines in the Extracellular Loops of the ATP Binding Cassette Transporter Pdr11p
Source: J Fungi (Basel). 2020 Dec 22;7(1):2. doi: 10.3390/jof7010002 (PMC7822014; doi:10.3390/jof7010002)
Supplement: Supplementary file 1 [file jof-07-00002-s001.pdf]

Functional significance of conserved cysteines in the extracellular loops of the ATP binding cassette transporter Pdr11p

Lyubomir Dimitrov Stanchev[1,2], *Magdalena Marek*[2,4], *Feng Xian*[3], *Mara Klöhn*[1], *Daniele Silvestro*[2], *Gunnar Dittmar*[3], *Rosa L. López-Marqués*[2], *Thomas Günther Pomorski*[1,2]

Supplementary Information

This file includes all supplementary tables S1-S4 and supplementary figure S1 for the manuscript.

Supplementary Table S1. Yeast strains used in this study.

Strain	Genotype	Source
W303	*MATα ade2-1 his3-11,15 leu2-3,112 trp1-1 ura3-1 can1-100*	Roland Lill, Philipps Universität Marburg
hem1Δ	W303 *hem1Δ::LEU2*	[13]
hem1Δaus1Δpdr11Δ	W303 *hem1Δ::LEU2 pdr11Δ::loxP aus1Δ::loxP-HIS5Sp-loxP*	[13]
hem1Δaus1Δpdr11Δ-PDR11	W303 *hem1Δ::LEU2 pdr11Δ::loxP aus1Δ::loxP-HIS5Sp-loxP ura3Δ::caURA3 ADH1-FLAG-PDR11-GFP*	This study
hem1Δaus1Δpdr11Δ-PDR11^{C582S}	W303 *hem1Δ::LEU2 pdr11Δ::loxP aus1Δ::loxP-HIS5Sp-loxP ura3Δ::caURA3 ADH1-FLAG-PDR11^{C582S} –GFP*	This study
hem1Δaus1Δpdr11Δ-PDR11^{C603S}	W303 *hem1Δ::LEU2 pdr11Δ::loxP aus1Δ::loxP-HIS5Sp-loxP ura3Δ::caURA3 ADH1-FLAG-PDR11^{C603S} –GFP*	This study
hem1Δaus1Δpdr11Δ-PDR11^{C1290S}	W303 *hem1Δ::LEU2 pdr11Δ::loxP aus1Δ::loxP-HIS5Sp-loxP ura3Δ::caURA3 ADH1-FLAG-PDR11 ^{C1290S} –GFP*	This study
hem1Δaus1Δpdr11Δ-PDR11^{C1306S}	W303 *hem1Δ::LEU2 pdr11Δ::loxP aus1Δ::loxP-HIS5Sp-loxP ura3Δ::caURA3 ADH1-FLAG-PDR11 ^{C1306S}–GFP*	This study
hem1Δaus1Δpdr11Δ-PDR11^{C1330S}	W303 *hem1Δ::LEU2 pdr11Δ::loxP aus1Δ::loxP-HIS5Sp-loxP ura3Δ::caURA3 ADH1-FLAG-PDR11^{C1330S}–GFP*	This study
hem1Δaus1Δpdr11Δ-PDR11^{C1333S}	W303 *hem1Δ::LEU2 pdr11Δ::loxP aus1Δ::loxP-HIS5Sp-loxP ura3Δ::caURA3 ADH1-FLAG-PDR11 ^{C1333S}–GFP*	This study

Supplementary Table S2. Plasmids used in this study.

Plasmids	Template/Purpose	Source
pESC-URA-FLAG-PDR11		[9]
pESC-URA-FLAG-PDR11-GFP		[9]
pESC-URA-FLAG-PDR11^{C582S} -GFP	pESC-URA-FLAG-PDR11-GFP	This study
pESC-URA-FLAG-PDR11^{C603S}-GFP	pESC-URA-FLAG-PDR11-GFP	This study
pESC-URA-FLAG-PDR11^{C1290S}-GFP	pESC-URA-FLAG-PDR11-GFP	This study
pESC-URA-FLAG-PDR11^{C1306S}-GFP	pESC-URA-FLAG-PDR11-GFP	This study
pESC-URA-FLAG-PDR11^{C1330S}-GFP	pESC-URA-FLAG-PDR11-GFP	This study
pESC-URA-FLAG-PDR11^{C1333S}-GFP	pESC-URA-FLAG-PDR11-GFP	This study
pESC-URA-FLAG-PDR11^{C582S}	pESC-URA-FLAG-PDR11	This study
pESC-URA-FLAG-PDR11^{C603S}	pESC-URA-FLAG-PDR11	This study
pESC-URA-FLAG-PDR11^{C1290S}	pESC-URA-FLAG-PDR11	This study
pESC-URA-FLAG-PDR11^{C1306S}	pESC-URA-FLAG-PDR11	This study
pESC-URA-FLAG-PDR11^{C1330S}	pESC-URA-FLAG-PDR11	This study
pESC-URA-FLAG-PDR11^{C1333S}	pESC-URA-FLAG-PDR11	This study
pVW126-pCYC	Vector backbone	[10]
pSIV340-pADH1	Amplification of *ADH1* promoter	[10]
pVW126-pCYC-FLAG-PDR11-GFP	pESC-URA-FLAG-PDR11-GFP	This study
pVW126-pADH1-FLAG-PDR11-GFP	pESC-URA-FLAG-PDR11-GFP	This study
pVW126-pADH1-FLAG-PDR11^{C582S} -GFP	pESC-URA-FLAG-PDR11^{C582S}-GFP	This study
pVW126-pADH1-FLAG-PDR11^{C603S}-GFP	pESC-URA-FLAG-PDR11^{C603S}-GFP	This study
pVW126-pADH1-FLAG-PDR11^{C1290S}-GFP	pESC-URA-FLAG-PDR11^{C1290S}-GFP	This study
pVW126-pADH1-FLAG-PDR11^{C1306S}-GFP	pESC-URA-FLAG-PDR11^{C1306S}-GFP	This study
pVW126-pADH1-FLAG-PDR11^{C1330S}-GFP	pESC-URA-FLAG-PDR11^{C1330S}-GFP	This study
pVW126-pADH1-FLAG-PDR11^{C1333S}-GFP	pESC-URA-FLAG-PDR11^{C1333S}-GFP	This study

Supplementary Table S3. Oligonucleotides used in this study

Name	Oligonucleotide	Purpose
F_ADH1<br>R_ADH1	GGGCGAATTGGAGCTCATCCTTTTGTTGTTTCCGGG<br>CATCCTTGTAATCCATAGTTGATTGTATGCTTGGTAT	Amplification of *pADH1* from pSIV340 to clone in pVW126
F_pSIV3<br>R_pSIV2	ATGGATTACAAGGATGACGA<br>ACTCGAGTTAGGATCCTTACTTGTACAGCTCGTCCATG	Amplification of FLAG-PDR11*-GFP cassettes from pESC-URA- FLAG-PDR11*-GFP for cloning into pVW126
F_C1<br>R_C1	ACCTGAAACTAGAC**aGT**CATGAAAGTATTATTCC<br>GGAATAATACTTTCATG**ACt**GTCTAGTTTCAGGT	Mutagenesis of *PDR11*^{C582S}
F_C2<br>R_C2	AGTCATAAAGCT**aGT**GCCTGGCAAGGC<br>GCCTTGCCAGGC**ACt**AGCTTTATGACT	Mutagenesis of *PDR11*^{C603S}
F_C3<br>R_C3	AGAGAAGTAAAC**aGC**TCGACAAGTGAA<br>TTCACTTGTCGA**GCt**GTTTACTTCTCT	Mutagenesis of *PDR11*^{C1290S}
F_C4<br>R_C4	ATGGGTCAAACC**aGC**GGTCAGTTTATG<br>CATAAACTGACC**GCt**GGTTTGACCCAT	Mutagenesis of *PDR11*^{C1306S}
F_C5<br>R_C5	ACATACACCGTC**aGC**GCCTACTGCATGT<br>ACATGCAGTAGGC**GCt**GACGGTGTATGT	Mutagenesis of *PDR11*^{C1330S}
F_C6<br>R_C6	GTCTGCGCCTAC**aGC**ATGTACACTGTT<br>AACAGTGTACAT**GCt**GTAGGCGCAGAC	Mutagenesis of *PDR11*^{C1333S}
Cys1-2_F<br>Cys1-2_R	ATTTTTGCATGGTGTCACTATTTGCATTAAC<br>AAGGGACAGTATCTTTTGAGATAGTTATTCCAG	Sequencing of *PDR11*^{C582S} and *PDR11*^{C603S} integrated in the chromosome to confirm introduced point mutations
Cys3-6_F<br>Cys3-6_R	ATTTGGGTTGTGGCTTCTATATTCTGC<br>CCATCAATCACAGAAGGCCAAATTTTTTTGA	Sequencing of *PDR11*^{C1290S}, *PDR11*^{C1306S}, *PDR11*^{C1330S} and *PDR11*^{C1333S} integrated in the chromosome to confirm introduced point mutations
Int_F<br>Int_R	ATGGACGAGCTGTACAAGTAAGG<br>TTGGAACTCTTGTTGTTCTTTGGA	Confirmation of FLAG-PDR11*-GFP cassettes integration in the chromosome
Int2_F<br>Int2_R	AAGTTATCTGATGTAGAAAAGGATTAAAGATG<br>ACCAATATATTGTTTCTTTACGGTATTACC	Confirmation of FLAG-PDR11*-GFP cassettes integration in the chromosome

*Wild type *PDR11* or single cysteine mutants *C582S, C603S, C1290S, C1306S, C1330S or C1333S*

#Mutated nucleotides are indicated in lowercase. Changed amino acids have codons indicated in boldface type.

Supplementary Table S4. List of targeted peptides for LC-MS/MS analysis

Mass [m/z]	Charge state	Polarity	Start [min]	End [min]	NCE	Peptide sequence
848.399021	2	Positive	0	85	28	AC[+71.037114]AWQGATLGNDYVR (light)
841.391196	2	Positive	0	85	28	AC[+57.021464]AWQGATLGNDYVR (light)
389.543655	3	Positive	0	85	28	LDSHESIIPR (light)
418.548412	3	Positive	0	85	28	LDC[+71.037114]HESIIPR (light)
413.876528	3	Positive	0	85	28	LDC[+57.021464]HESIIPR (light)
804.891886	2	Positive	0	85	28	ASAWQGATLGNDYVR (light)
1255.241873	3	Positive	0	85	30	EVNSSTSEMVPSQPVMGQTC[+71.037114]GQFMKPFIDEFGGK (light)
1250.56999	3	Positive	0	85	30	EVNSSTSEMVPSQPVMGQTC[+57.021464]GQFMKPFIDEFGGK (light)
1255.241873	3	Positive	0	85	30	EVNC[+71.037114]STSEMVPSQPVMGQTSGQFMKPFIDEFGGK (light)
1250.56999	3	Positive	0	85	30	EVNC[+57.021464]STSEMVPSQPVMGQTSGQFMKPFIDEFGGK (light)
1284.24663	3	Positive	0	85	30	EVNC[+71.037114]STSEMVPSQPVMGQTC[+71.037114]GQFMKPFIDEFGGK (light)
1279.574746	3	Positive	0	85	30	EVNC[+71.037114]STSEMVPSQPVMGQTC[+57.021464]GQFMKPFIDEFGGK (light)
1279.574746	3	Positive	0	85	30	EVNC[+57.021464]STSEMVPSQPVMGQTC[+71.037114]GQFMKPFIDEFGGK (light)
1274.902863	3	Positive	0	85	30	EVNC[+57.021464]STSEMVPSQPVMGQTC[+57.021464]GQFMKPFIDEFGGK (light)

Supplementary Figure

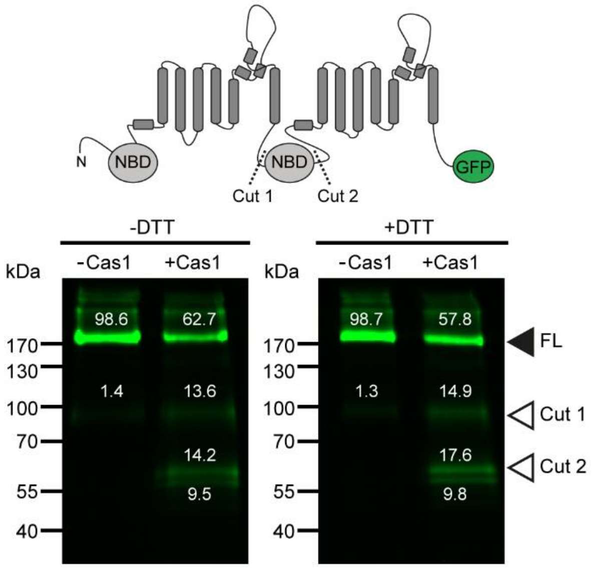

Supplementary Figure S1. Analysis of Pdr11p-GFP after limited proteolysis under reducing or non-reducing conditions. Purified full-length (FL) Pdr11p-GFP (filled arrowhead) was incubated in the absence (-Cas1) and presence (+Cas) of caspase-1 and subsequently analysed after SDS-PAGE by in-gel fluorescence imaging. Before SDS-PAGE, the samples were incubated 30 min at 37°C in SDS loading buffer without (-DTT) or with (+DTT) 50 mM dithiothreitol. Cut 1 and Cut 2 (open arrowheads) correspond to caspase-1 recognition sites D724 and D876, respectively. Number insets indicate the relative GFP fluorescence intensity (%) for the protein bands in each lane. Results are representative of at least three independent experiments. NBD, nucleotide binding domain.
